# High-Resolution Iodine-Enhanced Micro-Computed Tomography of Intact Human Hearts for Detailed Coronary Microvasculature Analyses

**DOI:** 10.3390/jimaging10070173

**Published:** 2024-07-18

**Authors:** Joerg Reifart, Paul Iaizzo

**Affiliations:** Visible Heart® Laboratories, Institute for Engineering in Medicine, Department of Surgery, University of Minnesota, Minneapolis, MN 55455, USA

**Keywords:** coronary vasculature, 3D imaging, contrast agent, micro-CT, iodine, artery segmentation, chronic total occlusion

## Abstract

Identifying the detailed anatomies of the coronary microvasculature remains an area of research; one needs to develop methods for non-destructive, high-resolution, three-dimensional imaging of these vessels for computational modeling. Currently employed Micro-Computed Tomography (Micro-CT) protocols for vasa vasorum analyses require organ dissection and, in most cases, non-clearable contrast agents. Here, we describe a method developed for a non-destructive, economical means to achieve high-resolution images of the human coronary microvasculature without organ dissection. Formalin-fixed human hearts were cannulated using venogram balloon catheters, which were then fixed into the specimen’s aortic root. The canulated hearts, protected by a polyethylene bag, were placed in radiolucent containers filled with insulating polyurethane foam to reduce movement. For vasculature staining, iodine potassium iodide (IKI, Lugol’s solution; 6.3% Potassium Iodide, 4.1% Iodide) was injected. Contrast distributions were monitored using a North Star Imaging X3000 micro-CT scanner with low-radiation settings, followed by high-radiation scanning (3600 rad, 60 kV, 900 mA) for the final high-resolution imaging. We successfully imaged four intact human hearts presenting with chronic total coronary occlusions of the right coronary artery. This imaging enabled detailed analyses of the vasa vasorum surrounding stenosed and occluded segments. After imaging, the hearts were cleared of iodine and excess polyurethane foam and returned to their initial formalin-fixed state for indefinite storage. Conclusions: the described methodologies allow for the non-destructive, high-resolution micro-CT imaging of coronary microvasculature in intact human hearts, paving the way for detailed computational 3D microvascular reconstructions with a macrovascular context.

## 1. Introduction

There is ongoing interest in understanding the detailed anatomies of the healthy and diseased coronary microvasculature, i.e., both the perivascular vasa vasorum and the downstream perfusing microvasculature (<300 μm). The coronary vasa vasorum are of interest relative to their roles in atherosclerotic processes. At the same time, the distal myocardium perfusing microvasculature has become of clinical interest because its dysfunction is of therapeutic importance. Microvascular dysfunction leads to symptoms of chest pain but also indicates a higher risk of major adverse cardiac events [1,2,3]. Furthermore, existing collaterals, plaque morphologies, and potential microchannels, which are of interest in the treatment of chronic total coronary occlusions, have not been studied in intact human hearts [4,5]. The growing uses of microvascular dysfunction testing and new clinical tools like contrast-enhanced ultrasound (CEUS) and superb microvascular imaging (SMI) stress the clinical need for a better understanding of the coronary microvasculature as part of the entire coronary vascular tree [6].

Micro-computed tomography (micro-CT) is a powerful non-destructive imaging technique that has been used to achieve high-resolution three-dimensional scans of coronary arteries using different contrast media [2,7,8,9]. MV-122/MV-120 (Microfil^®^, Flow Tech, Inc., Carver, MA, USA); the most widely used contrast medium to scan microvasculature yields reliable results but will irretrievably stay in the tissue since it polymerizes [2,7,10,11]. Recently, iodine-based contrast, as described by Self et al., was shown to result in stained vessels without remaining within the tissue, even allowing plaque morphology differentiation [8,9,12,13]. However, all previously described methods require the dissection of the artery from the specimen, sometimes embedding the artery in paraffin, thereby taking it out of its microvascular context. Previously, scanning entire hearts at microvascular resolution has not been successful. The primary reason this has not been done before was the lack of methods to mitigate tissue movement (due to relaxation and dehydration), resulting in blurred images [14].

To overcome this, we developed an iodine-enhanced high-resolution micro-CT method to examine the coronary microvasculature in intact perfusion-fixed human hearts. The contrasting technique used is a variation of the well-described technique with iodine potassium iodide (IKI, Lugol’s solution) applied to coronary vasculature [15]. Our approach aligns with the growing need for advanced imaging techniques to facilitate a deeper understanding of micro- and macrovascular pathologies in the context of cardiovascular diseases.

## 2. Materials and Methods

Human hearts were procured through the Anatomy Bequest Program at the University of Minnesota or from LifeSource, Inc. (St. Paul, MN, USA), for which consent was obtained to use these organs for research purposes. 

Deidentified clinical data, such as coronary artery status, were conveyed at the time of donation. When the hearts were initially received, the fresh organs were pressure–perfusion fixed with a 10% formalin solution for blood clearance, as previously described [16]. We selected hearts from the Visible Heart^®^ Human Heart Library that were known to have coronary artery disease and had high likelihoods of a chronically occluded artery (e.g., previous coronary artery bypass grafts or multiple risk factors). An intracoronary contrast injection was performed under fluoroscopy to confirm the presence of a chronic total occlusion. To reduce formalin exposure, specimens were rinsed in water for 24 h before these studies.

For selective contrast injection, coronary ostia were visualized using an endoscopic camera (OLYMPUS iPLEX NX, IV9435N, Olympus Corporation, Shinjuku, Tokyo, Japan) within the aorta. Venogram catheters (Attain 6125-18, Medtronic, Minneapolis, MN, USA) with an occluding balloon at the distal end were inserted and blocked with 1.5 mL air to prevent back-flow and enable antegrade contrast injections. 

Using isolated porcine hearts left over from other research, the methods were developed and refined over ten months, trialing different techniques to improve image clarities by reducing movement artifacts (viscoelastic polyurethane-foam cut-outs, tape fixation, freezing, and rigid polyurethane foam casting were employed), as well as utilizing different contrasting techniques (Microfil® (Microfil®, Flow Tech, Inc., Carver, MA, USA), Barium sulfate solution (Barium sulfate solution, Sigma-Aldrich, B3758, St. Louis, MO, USA), Iohexol (Iohexol, GE Healthcare, Chalfont St. Giles, UK), and iodine potassium iodide (Iodine potassium iodide, Carolina Biological Supply Company, 87-2797, Burlington, NC, USA)). 

**Scheme 1:**
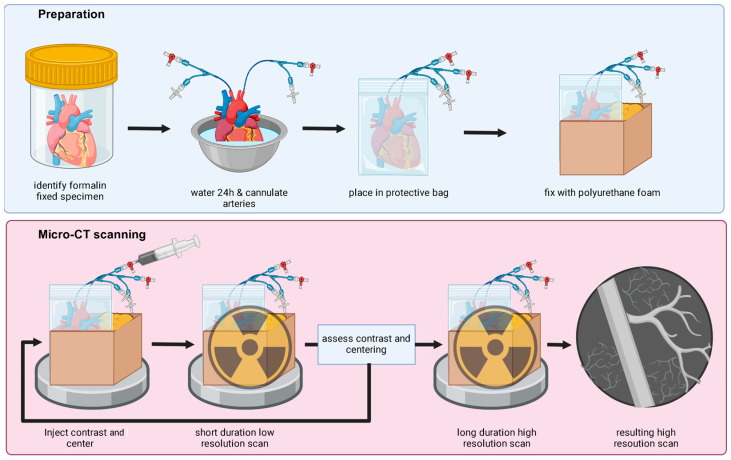
Overview of the employed methodologies: In the preparation phase, the heart is removed from formalin and watered for 24 h. Before being placed in a sealable bag, the coronary arteries are canulated. The specimen is then placed in a box filled with radiolucent hardening polyurethane foam. In the scanning phase, the heart is placed on the scanner’s rotary plate; contrast is administered, and repeated short-duration, low-resolution scans are used to assess the position and the contrast distribution. Once this step is complete, a resolution scan is performed.

To mitigate tissue movements, we placed the given heart into a sealed polyethylene bag except for exiting catheters. The heart was put into a 13 × 13 × 19 cm radiolucent container (cardboard/Styrofoam). Next, hardening polyurethane foam (insulation foam) was injected into the box to fix the heart three-dimensionally (Scheme 1).

Iodine potassium iodide (IKI) as aqueous solution was used as a diffusible radiopaque contrast agent (Lugol’s solution, potassium iodide 6.3%, iodine 4.1%, Carolina Biological Supply Company, 87-2797, Burlington, NC, USA). Repeatedly, 15 mL of undiluted solution was manually injected with slow and steady pressure. After each injection, micro-CT scans using a North Star Imaging X3000 Micro-CT scanner (NorthStar Imaging, Rogers, MN, USA) with a low number of projections (360 rad over 6–12 min) were performed to verify contrast distribution within the regions of interest and to initially center the hearts. Once sufficient vascular contrast was reached, the coronary arteries were flushed with regular saline solution to improve lumen contours.

Subsequently, high-resolution scans at 3600 rad, taking 60 min each, were performed (Table 1). A short focal distance to the radiation source was chosen, optimizing image resolutions without having the specimen come into contact with the X–ray emitter.

Images were reconstructed using NSI efx-CT software (North Star Imaging, Rogers, MN, USA) and then exported to DICONDE, TIFF, or JPEG format. Contrast-to-noise ratios (CNR) were calculated for all scans, placing a region of interest within the contrasted coronary artery wall (contrast) and filled air, non-contrasted ventricle cavity (noise). Results are presented as mean ± standard deviation.

All image volumes were then loaded into MIMICS (26.0, Materialise, Plymouth, MI, USA) for greyscale-threshold-based segmentations and further analyses. Variable photon attenuation line profiles were generated from cross-sectional cuts through the artery walls, analogous to previous reports [9]. 

Three-dimensional reconstructions with masks of each vasa vasorum, vessel adventitia, media, calcium, and lumen were created using semi-automatic segmentation tools with adaptive region-based thresholding.

Following staining and scanning, the hearts were carefully unpacked, using acetone, to soften the hard-to-remove parts of the employed polyurethane foam. The hearts were then taken out of their polyethylene bags and rinsed in water for 2–4 weeks, after which they underwent micro-CT scanning again to confirm contrast clearance before being returned to formalin.

## 3. Results

### 3.1. Imaging Outcomes and Generated Models

Four different hearts were successfully studied after establishing the contrast and micro-CT protocols. Injections of 110 ± 48 mL of the IKI solution lead to sufficient contrast amounts and equal diffusions. Multiple centering scans and contrast injections were necessary until the desired image qualities and artery positions were obtained, typically taking 9–12 h (9 ± 1.5 h) over a two-day period. The resulting scanning sets encompassed a tissue volume of 15 × 30 × 50 mm 3600 DICOM images (21.6 GB per scan). Whole heart scanning via merged volume from multiple images (MosaiX (setting on NSI X3000), NorthStar Imaging, Rogers, MN, USA) required scan times of 16 h, with subsequent project sizes exceeding 120 GB per scan.

**Figure 1:**
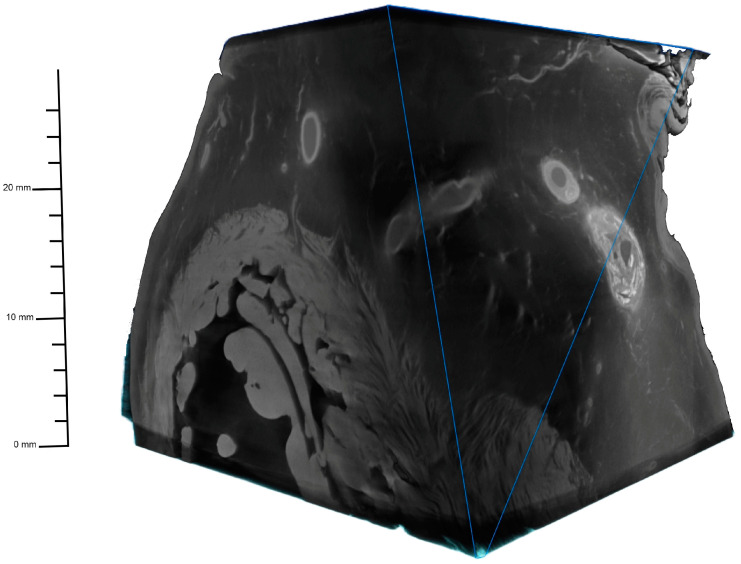
Shown here is an example of iodine diffusion enhanced high-resolution micro-computed tomography of the intact human heart, presenting with selective contrasting of a severely stenotic right coronary artery. The generated image reconstruction was created utilizing the NSI efx-CT software version 2.3.5.4.

**Figure 2:**
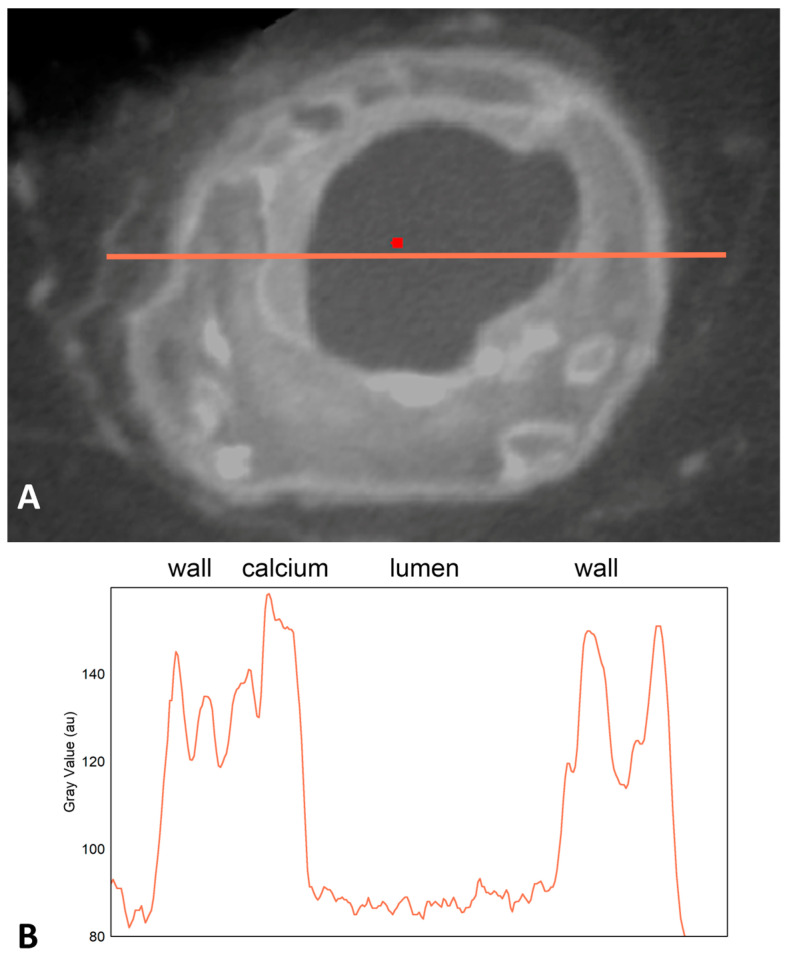
(**A**) Shows a cross-section of the proximomedial right coronary artery. The orange line indicates the vessel wall line profile in (**B**). One can observe the increased values at the outer and inner borders, delineating intima and adventitia, note the higher attenuation in calcified plaques. Beam hardening artifacts in solid calcified plaque often caused inaccurate representations mimicking a hypodense center.

The resulting average isotropic voxel resolution was 20.7 ± 0.7 μm with an average contrast-to-noise ratio of 22 ± 4.9 dB, allowing us to effectively visualize the coronary arteries, the vessel walls, and the surrounding microvasculature, all in detail without motion artifacts (blurring); see Figure 1. Extensive calcified plaques induced beam hardening artifacts; see Figure 2.

**Figure 3:**
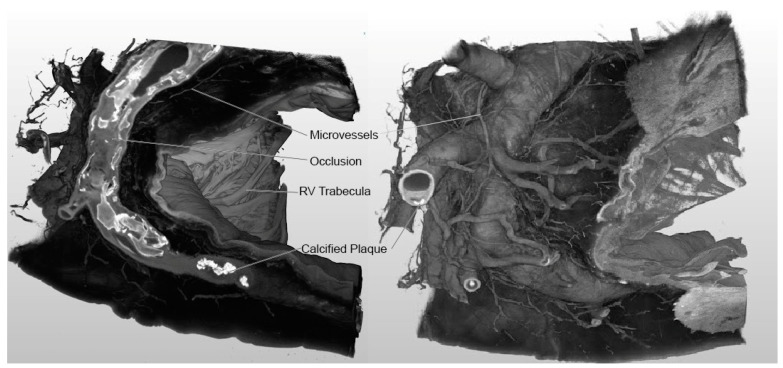
Presented here are the combined non-segmented 2D and 3D representations of a severely diseased right coronary artery with an occlusion in the medial segment. These observations highlight the employed methodologies being capable of discerning even micro vessels < 300 μm, assessing plaque composition and appreciating the anatomical context along the right ventricle.

Both the 2D images and the 3D, fixed threshold-based, non-segmented reconstructions showcased the intricate networks of the microvasculature, also highlighting any area of stenosis and occlusion (see Supplemental Video S1, Figure 3).

### 3.2. Individual Line Profiles

The obtained cross-sectional vessel wall line profiles from our iodine diffusion contrast staining with IKI elicited profile lines comparable to those published by Self et al. [9]. There were consistent increases in signal densities at the adventitia and more continuous values throughout the media. Intensity peaks in lipid-rich atherosclerotic plaques and calcium depositions in the vessel with atherosclerotic plaques were consistently observed (see Figure 2, Supplemental Video S2).

### 3.3. Vessel Segmentations

**Figure 4:**
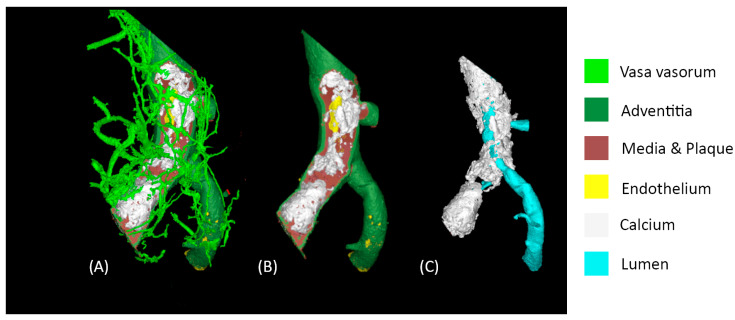
Shown here is a three-dimensional segmentation of the reconstructed occluded coronary artery segment; observe the intricate network of vasa vasorum (**A**), and the extensive calcium burden within the vessel, the media, plaque, and vessel walls (**B**). One can visualize the relationships to the calcified plaques within the lumen (**C**).

The contrast solution enhanced the artery’s layers (intima, adventitia) and the vasa vasorum (Figure 4), allowing segmentation using adaptive thresholding. As the principal vessel and the surrounding vasculature were contrasted, segmentation of collaterals and myocardium-perfusing arterioles was also possible (Supplemental Video S1).

### 3.4. Specimen Assessments Post-Processing

Repeat scanning after the provided contrast washout periods revealed that the studied perfusion-fixed heart specimens maintained structural integrities throughout these investigational processes. While providing critical visualizations during scanning, the contrast agents were cleared from the tissues after 2–4 weeks of rinsing.

## 4. Discussion

We present imaging methodologies for achieving high-contrast resolution imaging of human heart coronary vasculature without destroying the studied specimen. Both the perfusion-fixed heart preparation and staining techniques were aimed at preserving the macroscopical integrity for further usability of these specimens. We identified that utilizing an IKI (Lugol’s) solution is a cost-effective substitute for Iohexol for iodine-contrasted coronary micro-CT, and our methodologies for specimen preparation allow one to critically visualize intact coronary microvasculature.

Previously reported vessel-contrasting procedures often result in the contrast agents staying within the vessels. The most widely used product to visualize microvasculature with micro-CT has been the radiopaque, lead-containing, low-viscosity liquid MV-122 (Microfil^®^, Flow Tech, Inc., Carver, MA, USA) [2,7,10]. Typically, it has been described that MV-122 is injected into the artery, which is then embedded in low-melting-point paraffin wax to reduce motion artifacts; both procedures which permanently alter the specimen [7]. Noteworthy, low-viscosity barium-based contrast solutions (BaCl_2_/gelatin mixture) are an alternative to MV-122 [17]. Yet, our experiments leading up to the development of the Lugol-based technique indicated that barium-based solutions could not be fully cleared from the given specimen after scanning.

A similar method to the one we describe here is the reported iodine-based technique, which utilizes an iodine-enhanced micro-CT approach using Iohexol. Just like IKI, Iohexol stains the vessel wall as well as the surrounding tissues. This results in imaging of the vessel wall, showing vessel wall thickness and plaque compositions as opposed to the lumenogram of other methods [8,9,13].

The Lugol’s solution we employed in our study provided equivalent image resolutions and line profiles at lower costs than Iohexol, which can be up to 30 times the price of commercially available Lugol’s. To date, with its approximated 200 years of uses in medicine, Lugol’s solution is ubiquitous and affordable, whereas the availability of iodinated intravenous contrast solutions has recently been difficult and even unreliable [18,19]. Previously reported needs for high concentrations or long staining times of certain specimens did not apply well to the needed staining of the cardiac vasculature via intraarterial injection. Furthermore, total heart specimen immersion would require much greater volumes [20]. In comparison to Iohexol 250 mg/L, which has a viscosity of approximately 5 mPa×s at 25 degrees Celsius, Lugol’s solution has a lower estimated viscosity of 1 mPa×s; this in turn enables it to penetrate into the microvasculature more readily [21,22].

It should be noted that the iodine and potassium iodide concentrations can vary significantly between contrast manufacturers, and some manufacturers do not disclose their exact contents; hence, often researchers prefer custom-mixed solutions, which gives complete control over the respective concentrations [15,20].

Notable results obtained from our high-resolution 3D scanning of coronary microvasculature in relation to their macrovascular context may aid us in the following ways:

(a) Enhancing our understanding of collateral coronary anatomy and potential recruitment in diseased or fully occluded coronary arteries. With the generated high-resolution 3D vasculature models, one can then employ computational fluid dynamics (CFD) analyses to simulate local hemodynamics and endothelial shear stresses [4,23,24].
(b) Developing novel devices, based on three-dimensional tissue density patterns within occluded segments, to increase future successes of interventional procedures to treat chronic total occlusions [25].
(c) More critically investigating vasa vasorum densities within artery segments associated with different disease states to contribute to a better understanding of atherogenesis and what makes coronary plaques vulnerable [2,7,26].
(d) Enabling novel explorations of the presence or absence of continuous intralesional microchannels that may present in a heart with true chronic total occlusions, including their complex and highly variable three-dimensional paths. Previous work on these anatomies has been limited to cross-sectional pathology/HE slices or rabbit animal models of the femoral artery [5].
(e) Being a part of the translational research needed to visualize structural microvascular dysfunctions, with the potential to identify predictive angiographic patterns [1,3].

### Potential Study Limitations

Keeping the complex anatomic structures of interest, in this case, the microvasculature of the coronary arteries, intact had previously posed challenges for obtaining high-resolution images. Scanning the right coronary artery will, in most cases, result in better-contrasted images since it is more likely to be embedded in pericardial fat, which is less dense than myocardium. Generally, a higher resolution is achieved the closer the object is to the micro-CT scanner’s tube, yet the given size of the heart specimen and the packing box posed some limits here. To reach high-resolution images below an isotropic voxel resolution of 40 μm, other non-conventional techniques not employed in our study, like Sub-Pixel processing and Limited Angle Tomography, can be attempted. We also did not perform any post-processing (e.g., AI-assisted denoising or filters). These techniques are known to lead to a better contrast-to-noise ratio [27,28]. Future improvements in CT technology, like wider access to and improvement of hierarchical phase-contrast tomography, as performed by the European Synchrotron Radiation Facility (ESRF)’s Extremely Brilliant Source (EBS), will also allow whole heart scanning at even higher resolutions [29].

We noted that beam hardening artifacts caused by coronary calcium can pose issues during subsequent three-dimensional segmentation, potentially leading to under-estimations of calcified plaque volumes; i.e., the inside of calcified plaque may become indistinguishable from soft plaque. It is possible that this problem can be improved by further refining our employed techniques: using copper filters, hardening the radiation beam, taking a native vessel scan before contrast injection (this artifact is not as pronounced in non-contrasted tissues), and/or employing advanced computational techniques like virtual dual-energy subtraction [30].

Lastly, the technique presented here can, to some degree, be very time-consuming, which could limit its adoption, though further refinements of our methodologies could reduce preparation and scanning times. For example, injecting a standardized volume (e.g., 150 mL) of contrast and subsequent multi-panel (MosaiX, NorthStar Imaging, Rogers, MN, USA) scanning of the heart in its entirety could reduce the total project time. Finally, AI-assisted automated segmentation (such as MONAI (NVIDIA Corporation, Santa Clara, CA, USA) and NVIDIA Clara(NVIDIA Corporation, Santa Clara, CA, USA)) could be employed to aid in accelerating the translations of complex 3D anatomic image volumes to research findings.

## 5. Conclusions

The results of our study demonstrate the feasibility and effectiveness of the employed staining and specimen preparation/packing techniques to achieve 20 micrometer high-resolution micro-CT images for detailed anatomic analyses of the coronary microvasculature within intact human hearts. These developed and employed methodologies can provide one with the tools to obtain new understandings of the complexities of the coronary microvascular architecture and its alterations in the context of coronary artery disease. The resulting models of human coronary micro- and macrovasculatures will aid our clinical understanding of cardiovascular diseases and be of use to those developing next-generation coronary therapeutic technologies.

## Figures and Tables

**Table 1:** Utilized sample settings for the micro-CT, for the regular acquisitions for right coronary artery anatomies. Note that the minimum resolvable feature sizes can be smaller than the employed focal spot sizes due to the system’s overall design and image processing capabilities. Yet, scanner settings must be tailored to each specimen due to variabilities in the tissue densities and degrees of contrast uptake.

Micro-CT Technique	
Voltage (kV)	60
Current (micro-amperes)	900
Focal spot (microns)	54
Effective pixel pitch (mm)	0.01989
Resolution (microns)	19.83
Tube to detector (mm)	1323.609
Tube to object (mm)	207.34
Calculated ug (mm)	0.209
Frame rate (fps)	1
Projections	3600

## Data Availability

The raw data supporting the conclusions of this article will be made available by the authors upon request.

## Supplementary Materials

The following supporting information can be downloaded at: https://www.mdpi.com/article/10.3390/jimaging10070173/s1, Video S1: Micro-CT scans through the contrasted coronary artery, at an earlier stage of contrast infusion and viewed from cranial to caudal. The venogram-catheter tip can be appreciated in the initial images. Video S2: Micro-CT images presented as continuous slices through the chronic total occlusion of a right coronary artery. Vasa vasorum, calcific plaques, and hypodense areas can be observed in different segments of the scanning slices. Supplemental Methods. Supplemental Table S1: Micro-CT settings geared towards reaching 20 micrometer resolution while keeping the calculated unsharpness as low as possible.
