# Supplementary material for: High-Resolution Iodine-Enhanced Micro-Computed Tomography of Intact Human Hearts for Detailed Coronary Microvasculature Analyses
Source: J Imaging. 2024 Jul 18;10(7):173. doi: 10.3390/jimaging10070173 (PMC11278041; doi:10.3390/jimaging10070173)
Supplement: Supplementary file 1 [file jimaging-10-00173-s001.zip › Supplemental Methods.pdf]

**Supplement to Methods:**

Additional insights from technique development:
Settings with lower Ug and lower current at the same resolution (Suppl Table 1) will not yield similar tissue differentiation but will take considerably longer. Though tissue differentiation is mostly determined by Voltage (lower Voltage leading to better contrast), we found a higher current to be important even though more calculated unsharpness is introduced.

**Supplemental Table S1**

| Micro-CT Technique | |
|---|---|
| Voltage (kV) | 60 |
| Current (micro-amperes) | 370 |
| Focal Spot (microns) | 22.2 |
| Effective pixel pitch (mm) | 0.01943 |
| Resolution (microns) | 19.62 |
| Tube to detector (mm) | 1175.598 |
| Tube to object (mm) | 179.811 |
| Calculated Ug (mm) | 0.1229 |
| Frame Rate (fps) | 0.5 |
| Projections | 3600 |
| Acquisition time (min) | 240 |
